# Short-term clinical outcomes of cytoreductive radical prostatectomy combined with abiraterone-based androgen deprivation therapy in oligometastatic prostate cancer

**DOI:** 10.1097/MD.0000000000049826

**Published:** 2026-07-24

**Authors:** Bai Li, Qin Qin, Jinyu Liao, Li Li, Xianlong Wang, Peng Hao, Aiping Zhang, Guoqing Zhang, Chao Huang

**Affiliations:** a Department of Urology, Dazhou Dachuan District People’s Hospital (Dazhou Third People’s Hospital), Dazhou, Sichuan Province, China; b Dazhou Central Hospital, Dazhou, Sichuan Province, China.

**Keywords:** clinical outcomes, oligometastatic prostate cancer, pharmacotherapy, radical prostatectomy, short-term prognosis

## Abstract

This retrospective cohort study evaluated the clinical value and short-term prognostic outcomes of cytoreductive radical prostatectomy using the cytoreductive radical prostatectomy (CRP) technique combined with pharmacotherapy in patients with oligometastatic prostate cancer (OPC). Consecutive OPC patients treated between January 2023 and December 2023 were included. According to the actual treatment received, patients were assigned to a pharmacotherapy group (control) or a CRP technique plus pharmacotherapy group (study), with 47 patients in each group. All patients were followed for 24 months. Baseline demographics and clinical variables were compared between groups. Treatment response was assessed using Prostate Cancer Working Group 3 criteria. Serum prostate-specific antigen levels were recorded at baseline and at 2, 6, and 12 months. Quality of life, pain, and anxiety were evaluated using the European Organisation for Research and Treatment of Cancer Quality of Life Questionnaire-Core 30, visual analog scale, and generalized anxiety disorder-7, respectively. Complications were graded by the Clavien–Dindo classification. Survival outcomes were analyzed using Kaplan–Meier methods. Baseline characteristics were comparable between groups. The CRP combined with intensified systemic therapy showed a higher proportion of complete/partial response and a lower proportion of progression than the pharmacotherapy group. Between-group differences in prostate-specific antigen were evident at the 2-month assessment. Posttreatment European Organisation for Research and Treatment of Cancer Quality of Life Questionnaire-Core 30 scores were higher, whereas visual analog scale and generalized anxiety disorder-7 scores were lower, in the combined-treatment group. Complications were infrequent and limited to grade I events, with no between-group difference. No deaths occurred during follow-up; progression-free survival was longer in the combined-treatment group. Cytoreductive radical prostatectomy using the CRP technique combined with pharmacotherapy was associated with improved short-term disease control and progression-free survival without an apparent increase in complications in OPC.

## 1. Introduction

Prostate cancer, as a common malignant tumor of the male genitourinary system, with its metastatic lesions being the main cause of patient mortality.^[1]^ Oligometastatic prostate cancer (OPC), as a transitional state between localized and widespread metastasis, typically refers to a special type where imaging studies reveal ≤5 metastatic lesions limited to non-visceral organs such as lymph nodes and bones.^[2]^ Traditional beliefs suggest that systemic pharmacotherapy should be the primary approach for metastatic prostate cancer. However, in recent years, it has been discovered that a comprehensive model combining local and systemic treatments can significantly improve patients’ prognosis.^[3]^ Previous studies have shown that circulating tumor cells released from the primary lesion of prostate cancer are one of the sources of metastatic cancer cells. These metastatic cancer cells can circulate back to the primary lesion, where both the primary and metastatic lesions can provide a “seed” for new metastases.^[4]^ In the oligometastatic stage, cancer cells in the metastatic lesions mostly originate from the primary lesion, exhibiting high tumor cell clone homogeneity. In contrast, in the advanced metastatic stage, cancer cells in the metastatic lesions may come from early metastatic sites, leading to high tumor cell clone heterogeneity and increased invasiveness. The primary tumor can upregulate the expression of proteins related to tumor signaling pathways, activating the Src kinase family that promotes tumor cell proliferation, invasion, and angiogenesis, further facilitating distant tumor metastasis.^[5]^ Therefore, removing the primary tumor can eliminate the cell factors that promote metastasis within the tumor cells, block related tumor signaling pathways, restrict the proliferation of more invasive tumor cells, delay tumor progression, enhance the patient’s response to systemic treatment, and improve the prognosis of cancer patients. Cytoreductive radical prostatectomy (CRP), as the standard treatment for localized prostate cancer, has evolved technically from open surgery to minimally invasive procedures such as laparoscopic radical prostatectomy and robot-assisted radical prostatectomy surgeries.^[6]^ For OPC patients, CRP can reduce tumor burden by removing the primary lesion, decrease the secretion of pro-metastatic factors, and enhance the body’s sensitivity to systemic treatment. However, there are still limited researches on the application of CRP combined with pharmacotherapy in OPC. This analysis aims to systematically explore the application value of CRP combined with pharmacotherapy in OPC, focusing on evaluating its short-term prognostic indicators, to provide evidence-based support for optimizing the treatment strategies for metastatic prostate cancer.

## 2. Methods

### 2.1. Research objects

This study was approved by the Ethics Committee of Dazhou Central Hospitall (Approval No. 2025-DZCH-kz145). This retrospective cohort study included consecutive patients diagnosed with OPC who received treatment at our institution between January 2023 and December 2023 and the final follow-up date was December 2025. Patients were categorized according to the treatment actually administered: a pharmacotherapy-only group (control group) and a CRP technique combined with pharmacotherapy group (study group), with 47 patients in each group based on the prespecified sample size target. Eligibility criteria were defined prior to case identification. Inclusion criteria were: meeting diagnostic criteria for OPC; histopathological confirmation of prostate cancer; absence of contraindications to surgery at the time of treatment decision-making; and confirmation of ≤5 metastatic lesions based on imaging evaluation, including bone scintigraphy and computed tomography and/or magnetic resonance imaging. Exclusion criteria were: presence of other concurrent malignancies; severe dysfunction of major organs; incomplete clinical records or follow-up data that precluded outcome assessment; and documented mental disorders that could compromise clinical evaluation or adherence. The requirement for informed consent was waived because of the retrospective nature of the study.

### 2.2. Treatment protocol

Both groups of patients were followed up for a period of 24 months.

Control group adopted systemic therapy. Patients received abiraterone acetate (1000 mg orally once daily) in combination with prednisone (5 mg orally twice daily) and goserelin (3.6 mg subcutaneous injection every 28 days). Goserelin was initiated 2 weeks after the start of abiraterone therapy and continued throughout follow-up.

Study group adopted CRP technique combined with pharmacotherapy. The procedure was performed through an abdominal approach by the same group of urologists with extensive laparoscopic surgical experience. After general anesthesia, the patients were placed in a head-down position with the feet elevated (approximately 30° head-down tilt). A Fr18 urinary catheter (Bard, USA, CFDA (I) 20162660995) was inserted, and a small incision was made below the navel. An insufflation needle was inserted to create a pneumoperitoneum with CO_2_. A 10 mm trocar was then inserted for the laparoscope. Under direct visualization, 2 additional 10-mm trocars were placed bilaterally at the lateral borders of the rectus abdominis muscles, approximately 2 cm below the umbilical level. Furthermore, two 5-mm trocars were inserted bilaterally, each located about 2 cm from the anterior superior iliac spine. All laparoscopic surgical instruments were then introduced through these established access ports. The procedure involved dissecting the prostate and periprostatic fat, opening the pelvic fascia on both sides, dividing the puboprostatic ligaments, ligating the dorsal vein complex, confirming the position of the bladder neck with gentle traction on the catheter, opening the bladder neck while protecting the ureteral orifices, identifying the seminal vesicles posterior to the prostate, and dividing the vas deferens. The retzius space was opened, the lateral prostatic ligaments were divided, and the decision to preserve the neurovascular bundles was made based on preoperative imaging assessment. The urethra was divided at the apex of the prostate. The entire prostate and seminal vesicle tissue were completely excised. The bladder neck and urethra were then continuously sutured using a double-needle technique from the 6 o’clock direction. Standard pelvic lymph node dissection was performed, including obturator, internal iliac, and external iliac lymph nodes. Postoperatively, patients received the same systemic therapy regimen as the control group, consisting of abiraterone acetate (1000 mg orally once daily), prednisone (5 mg orally twice daily), and goserelin (3.6 mg subcutaneous injection every 28 days). Systemic therapy was continued according to institutional treatment protocols throughout the follow-up period.

### 2.3. Observational indicators

First, compare baseline data between the 2 groups. Second, compare the efficacy of the 2 groups according to the Prostate Cancer Working Group 3 (PCWG3) criteria, which include: complete response defined as the disappearance of all target lesions, absence of new lesions, and normalization of tumor markers, maintained for at least 4 weeks. Partial response defined as a ≥50% decrease in serum prostate-specific antigen (PSA) level from baseline. Stable disease defined as a decrease of <50% or an increase of <25%. Disease progression defined as an increase of ≥25%.^[7]^ Third, compare serum PSA levels between the 2 groups at baseline, 2, 6, and 12 months postoperatively. Fourth, compare the quality of life (assessed by the European Organisation for Research and Treatment of Cancer Quality of Life Questionnaire-Core 30 [EORTC QLQ-C30] scale),^[8]^ pain scores (assessed by the visual analog scale [VAS]),^[9]^ and anxiety levels (assessed by the generalized anxiety disorder-7 scale [GAD-7])^[10]^ before and after the intervention. The GAD-7 employs a 4-point Likert scale (0 = “Not at all,” 1 = “Several days,” 2 = “More than half the days,” 3 = “Nearly every day”), with a total score range of 0 to 21. The anxiety levels are categorized as follows: 0 to 4 = no anxiety, 5 to 9 = mild anxiety, 10 to 14 = moderate anxiety, and 15 to 21 = severe anxiety. The scale demonstrates good reliability and validity, with a Cronbach α coefficient of 0.898. Fifth, postoperative complications were assessed within 90 days after surgery. Complication data were collected through inpatient medical records, scheduled outpatient follow-up visits, and standardized telephone interviews. The actively monitored complications included urinary incontinence, urinary retention, lymphocele formation, postoperative bleeding, urinary tract infection, anastomotic leakage, wound complications, hospital readmission, and any other adverse events requiring medical intervention. All complications were classified and graded according to the Clavien–Dindo classification system. Sixth, compare the survival curves between the 2 groups, including overall survival and progression-free survival.

### 2.4. Statistical analysis

Experimental data collected were analyzed using Statistical Package for the Social Sciences (SPSS 27.0; International Business Machines Corporation). The Shapiro–Wilk test was used to assess normality. For normally distributed continuous data, results were presented as $\bar{X}\pm S$, and comparisons were made using independent sample *t* tests. Non-normally distributed data were presented as median with interquartile range *MQ*2 (*Q*1, *Q*3), and analyzed using the Mann–Whitney *U* test. Categorical data were presented as frequencies or rates, and comparisons were performed using χ^2^ test or Fisher exact test, with statistical significance set at *P* < .05. All analyses were performed according to the treatment actually received. Kaplan–Meier survival curves were constructed to estimate progression-free survival (PFS) and overall survival (OS). PFS was defined as the interval from treatment initiation to the 1st documented disease progression according to PCWG3 criteria or death from any cause, whichever occurred 1st. OS was defined as the interval from treatment initiation to death from any cause. Patients without progression or death at the last follow-up visit were censored on the date of their last clinical assessment. Survival distributions between groups were compared using the log-rank test. Hazard ratios and corresponding 95% confidence intervals were estimated using Cox proportional hazards regression models. Survival distributions between groups were compared using the log-rank test. Patients without progression or death at the last follow-up were treated as censored observations. Statistical significance was defined as a 2-sided *P* value < .05.

## 3. Results

### 3.1. Comparison of baseline data between 2 groups

There were no significant differences between the 2 groups in baseline characteristics such as age, height, weight, body mass index, hypertension, diabetes, smoking history, drinking history, and preoperative Gleason score (*P* > .05). The consistency of baseline data showed that the 2 groups of patients were highly comparable before treatment, excluding treatment differences that may be caused by different baseline characteristics, and providing a reliable basis for subsequent efficacy evaluation, as shown in Table 1.

**Table 1:** Comparison of baseline data between 2 groups.

Indicator		Control group (n = 47)	Study group (n = 47)	*t*/*χ*^2^ value	*P* value
Age (yr)		74.91 ± 5.64	72.66 ± 8.97	1.456	.149
Height (cm)		162.81 ± 7.10	163.49 ± 6.85	0.473	.638
Weight (kg)		61.06 ± 10.62	62.16 ± 9.03	0.541	.590
BMI (kg/m^2^)		23.06 ± 3.97	23.25 ± 2.99	0.262	.794
Hypertension	Yes	14 (29.79)	16 (34.04)	0.196	.658
	No	33 (70.21)	31 (65.96)		
Diabetes	Yes	5 (10.64)	7 (14.89)	0.382	.537
	No	42 (89.36)	40 (85.11)		
Smoking history	Yes	11 (23.40)	13 (27.66)	0.224	.636
	No	36 (76.60)	34 (72.34)		
Alcohol-drinking history	Yes	15 (31.91)	13 (27.66)	0.204	.652
	No	32 (68.09)	34 (72.34)		
Preoperative Gleason score (points)	>8	10 (21.28)	9 (19.15)	0.066	.797
	≤8	37 (78.72)	38 (80.85)		

BMI = body mass index.

### 3.2. Comparison of treatment efficacy between 2 groups

The efficacy of the study group patients was superior to that of the control group patients (*P* < .05). There were 28 cases in the study group with complete remission and 14 cases in partial remission, and 15 cases in the control group with complete remission and 10 cases in partial remission. The treatment plan in the study group performed better in promoting disease remission, which may be due to its more precise treatment strategy or stronger drug effect. This provides a basis for clinical selection of more effective treatment options, as shown in Table 2.

**Table 2:** Comparison of treatment efficacy between 2 groups.

	Number of cases	Complete response	Partial response	Stable disease	Disease progression
Control group	47	15 (31.91)	10 (21.28)	3 (6.38)	19 (40.43)
Study group	47	28 (59.57)	14 (29.79)	5 (10.64)	7 (14.89)
χ^2^ value		10.200			
*P* value		.017			

### 3.3. Comparison of PSA levels between 2 groups at preoperative, 2, 6, and 12 months after operation

The comparison of PSA levels between the 2 groups at baseline, 6 and 12 months after treatment showed no statistically significant differences (*P* > .05). At the 2-month assessment, the PSA level in the CRP plus pharmacotherapy group was higher than that in the pharmacotherapy-only group (2.35 vs 0.02, *P* < .05). Therefore, the 2-month PSA result did not indicate a superior early PSA decline in the combined-treatment group. This finding should be interpreted cautiously in the context of the retrospective study design, limited sample size, and potential variability in postoperative PSA assessment and systemic treatment response, as presented in Table 3.

**Table 3:** Comparison of PSA levels between 2 groups at preoperation, 2, 6, and 12 months after operation (ng/mL).

	Number of cases	Preoperation	2 months after operation	6 months after operation	12 months after operation
Control group	47	152.11 (61.12, 1049.55)	2.35 (0.35, 9.85)	0.06 (0.00, 0.25)	0.04 (0.04, 0.72)
Study group	47	83.21 (36.95, 317.73)	0.52 (0.32, 1.00)	0.46 (0.05, 2.47)	0.11 (0.02, 0.31)
*Z* value		−1.588	−2.640	−1.837	−0.336
*P* value		.112	.005	.066	.737

PSA = prostate-specific antigen.

### 3.4. Comparison of life quality, pain, and anxiety between 2 groups

The comparison of life quality, pain, and anxiety between the 2 groups of patients showed no statistically significant differences at baseline (*P* > .05). However, after treatment, the study group patients exhibited higher EORTC QLQ-C30 scores and lower scores on the VAS and GAD-7 compared to the control group (*P* < .05).The study group’s treatment plan is more effective in improving patients “quality of life and reducing pain and anxiety, which helps improve patients” treatment compliance and overall health status, as presented in Table 4.

**Table 4:** Comparison of life quality, pain, and anxiety between 2 groups.

Indicator		Control group (n = 47)	Study group (n = 47)	*t* value	*P* value
EORTC QLQ-C30	Before treatment	41.83 ± 6.19	41.67 ± 6.38	0.123	.902
	After treatment	52.82 ± 10.33	61.33 ± 10.37	3.986	<.001
VAS	Before treatment	5.38 ± 1.12	5.13 ± 1.21	1.039	.301
	After treatment	2.16 ± 0.92	1.31 ± 0.37	5.877	<.001
GAD-7	Before treatment	8.06 ± 2.37	7.73 ± 2.16	0.706	.482
	After treatment	3.79 ± 1.03	1.91 ± 0.88	9.514	<.001

EORTC QLQ-C30 = European Organisation for Research and Treatment of Cancer Quality of Life Questionnaire-Core 30, GAD-7 = generalized anxiety disorder-7, VAS = visual analog scale.

### 3.5. Comparison of complications between 2 groups

Postoperative complications were assessed within 90 days after surgery. Complication data were collected from inpatient medical records, scheduled outpatient follow-up visits, and standardized telephone interviews. The actively monitored complications included urinary incontinence, urinary retention, lymphocele formation, postoperative bleeding, urinary tract infection, anastomotic leakage, wound complications, hospital readmission, and any other adverse events requiring medical intervention. All complications were classified according to the Clavien–Dindo classification system.

Within the predefined 90-day assessment period, 2 grade I complications were identified in the CRP plus pharmacotherapy group, including 1 case of transient urinary incontinence managed conservatively and 1 case of urinary tract infection treated with oral antibiotics. In the pharmacotherapy-only group, 1 grade I adverse event related to medical treatment was observed. No cases of urinary retention, lymphocele formation, postoperative bleeding, anastomotic leakage, wound complications, hospital readmission, or Clavien–Dindo grade II or higher complications were recorded in either group. The overall complication rates did not differ significantly between the 2 groups, with 2/47 patients in the CRP plus pharmacotherapy group and 1/47 patient in the pharmacotherapy-only group experiencing adverse events. The overall complication rates did not differ significantly between the 2 groups (2/47 [4.3%] vs 1/47 [2.1%], χ^2^ = 0.344, *P* = .557).

### 3.6. Survival curve analysis of 2 groups

All patients completed the planned 24-month follow-up period, and the median follow-up duration was 24.0 months in both groups. No deaths occurred during follow-up, resulting in an overall survival rate of 100% in both groups. Therefore, comparative analysis of OS was not informative within the present short-term follow-up period.

**Figure 1.**
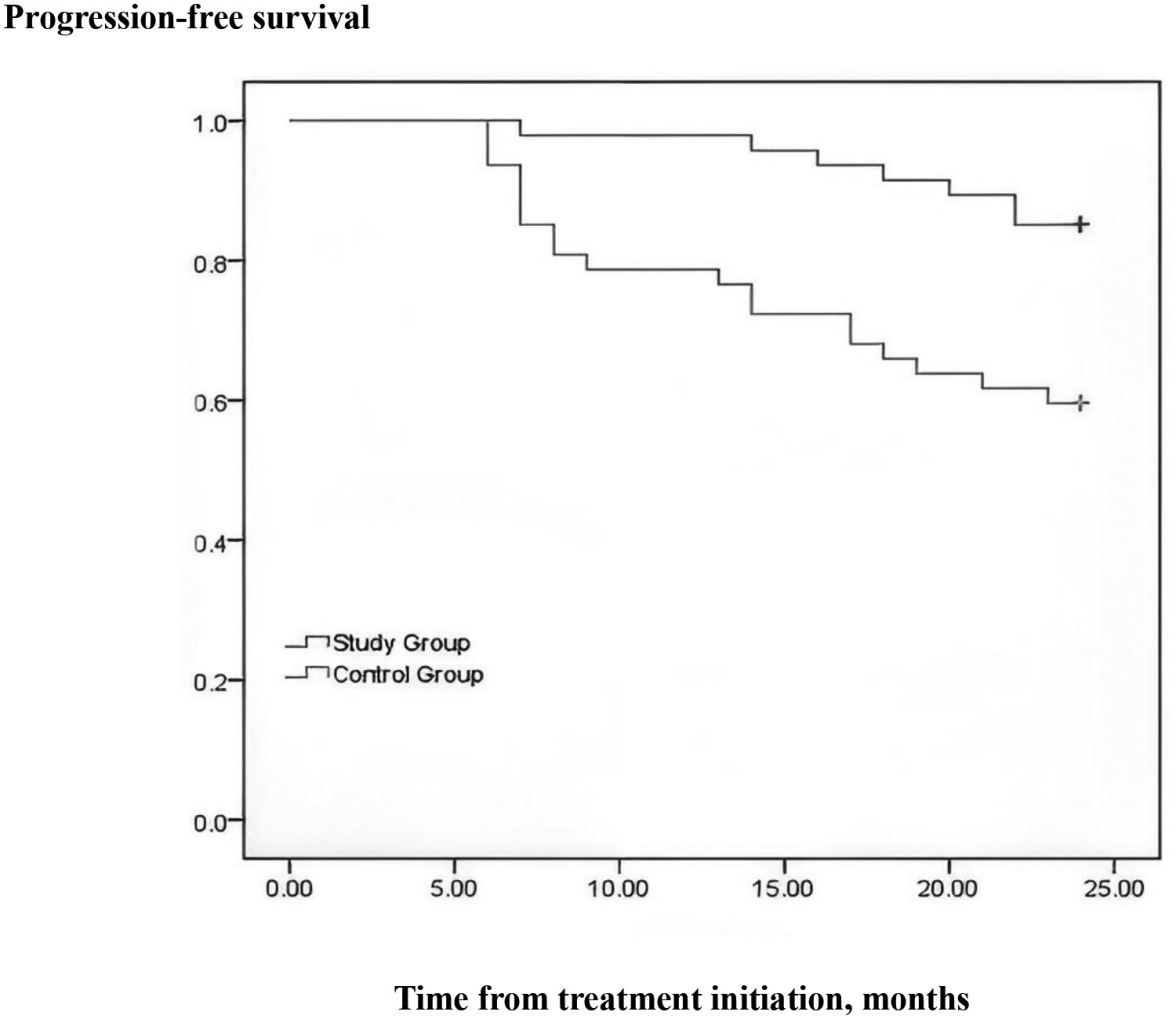
Kaplan–Meier curves for progression-free survival in patients with oligometastatic prostate cancer receiving pharmacotherapy alone or cytoreductive radical prostatectomy combined with pharmacotherapy. Patients without documented progression at the final follow-up visit were censored. Survival distributions were compared using the log-rank test (χ^2^ = 8.487, *P* = .004).

PFS was analyzed using the Kaplan–Meier method. Disease progression was defined according to PCWG3 criteria, and patients without documented progression or death at the final follow-up visit were censored. In the CRP plus pharmacotherapy group, 5 of 47 patients experienced disease progression, and 42 patients were censored without progression or death. In the pharmacotherapy-only group, 22 of 47 patients experienced disease progression, and 25 patients were censored without progression or death. The baseline number at risk was 47 patients in each group. Kaplan–Meier analysis demonstrated significantly prolonged PFS in the CRP plus pharmacotherapy group compared with the pharmacotherapy-only group (log-rank χ^2^ = 8.487, *P* = .004) (Fig. 1). Because complete individual patient-level event-time data were not preserved in the retrospective dataset, hazard ratios, 95% confidence intervals, and complete time-specific number-at-risk values could not be reliably reconstructed and were therefore not reported.

## 4. Discussion

This study aimed to investigate the application value of combined CRP technique and pharmacotherapy in OPC and focused on analyzing its short-term prognostic effects. The efficacy, quality of life, pain and anxiety levels, complication rates, and survival periods of patients with OPC were compared between pure pharmacotherapy and combined CRP technique and pharmacotherapy.

The results demonstrated that the efficacy of patients in the study group was superior to that of the control group. Specifically, a higher proportion of patients in the study group achieved complete or partial remission compared to the control group, while the proportion of patients with disease progression was lower. These findings support the effectiveness of the combined CRP technique and pharmacotherapy in OPC. Mechanistically, metastatic lesions in OPC often originate from the primary lesion, with high clonality of tumor cells. By surgically removing the primary lesion using the CRP technique, tumor burden can be reduced, leading to decreased secretion of pro-metastatic factors and increased sensitivity of the body to systemic therapy. Additionally, the presence of the primary lesion may promote the formation of new metastatic lesions by releasing circulating tumor cells. Removing the primary lesion can interrupt this process, thereby delaying tumor progression.^[11,12]^ Hence, the combined CRP technique and pharmacotherapy can more effectively control tumor growth and spread, increasing the remission rate of patients. PSA levels are crucial indicators for the diagnosis and prognosis assessment of prostate cancer. In this study, patients in the study group exhibited lower PSA levels at 2 months after operation compared to the control group, although there were no statistically significant differences in PSA levels at baseline and at 6 and 12 months after operation. This outcome suggests that the combined CRP technique and pharmacotherapy can more effectively reduce PSA levels in the short term, reflecting quicker control of tumor burden. The rapid decline in PSA levels may be associated with the removal of the primary lesion, which is a major source of PSA production. Removing the primary lesion can rapidly decrease PSA production.^[13,14]^ Furthermore, combined pharmacotherapy may further lower PSA levels by inhibiting the androgen receptor signaling pathway. However, over time, the difference in PSA levels between the 2 groups gradually diminishes, possibly due to the sustained effects of pharmacotherapy. Nevertheless, the early postoperative reduction in PSA levels is crucial for assessing treatment response and adjusting treatment strategies.^[15]^

The study further revealed that posttreatment, patients in the study group exhibited higher EORTC QLQ-C30 scores and lower scores on the VAS and GAD-7 compared to the control group. This indicates that the combined CRP technique and pharmacotherapy not only more effectively controls the tumor but also improves patients’ quality of life, reduces pain, and alleviates anxiety. The improvement in quality of life may be associated with enhanced tumor control efficacy. Delaying tumor progression and alleviating symptoms enable patients to better maintain daily activities and social functions. Additionally, the reduction in pain and anxiety may be linked to the synergistic effects of primary lesion removal and pharmacotherapy. The presence of the primary lesion can induce pain and discomfort through nerve compression and inflammatory reactions, and removing the primary lesion can alleviate these symptoms.^[16]^ Simultaneously, pharmacotherapy, by inhibiting tumor growth and metastasis, further alleviates the psychological burden on patients. In the study, 2 cases of Grade I complications occurred in the study group, while 1 case occurred in the control group. There were no Grade II or higher complications in either group, and the complication rates did not show any statistically significant differences. This suggests that the combined CRP technique and pharmacotherapy is as safe as pure pharmacotherapy and does not increase the risk of severe complications. The CRP technique, as a mature minimally invasive surgery, has a low complication rate.^[17]^ In this study, the surgeries were performed by experienced urologists, further reducing the surgical risks. Additionally, the combined pharmacotherapy did not increase complications, possibly due to the selection of drugs and dosage adjustments. Abiraterone, commonly used drugs for prostate cancer treatment, are widely recognized for their safety. Therefore, the feasibility of the combined CRP technique and pharmacotherapy in terms of safety is established.^[18]^

No patient deaths were observed during the follow-up period, resulting in a 100% overall survival rate for both groups. However, Kaplan–Meier survival analysis indicated that the study group had a longer progression-free survival time compared to the control group. This suggests that the combined CRP technique and pharmacotherapy can delay disease progression and improve patients’ progression-free survival period. The extension of the progression-free survival period may be attributed to the synergistic effects of primary lesion removal and pharmacotherapy. The removal of the primary lesion reduces tumor burden, decreases pro-metastatic factor secretion, and enhances the body’s sensitivity to systemic therapy. Pharmacotherapy further inhibits the proliferation and metastasis of tumor cells by suppressing the androgen receptor signaling pathway. Consequently, the combined CRP technique and pharmacotherapy can more effectively control tumor growth and spread, delaying disease progression.^[19]^

The results of this study indicate that the combined CRP technique and pharmacotherapy have significant application value in OPC. This approach not only effectively controls tumor growth and spread, improves patients’ remission rates and progression-free survival period, but also enhances quality of life, reduces pain, and alleviates anxiety. Furthermore, this approach is as safe as pure pharmacotherapy and does not increase the risk of severe complications. From an economic perspective, the combined CRP technique and pharmacotherapy may reduce hospitalization time and medical costs, ultimately lowering overall healthcare expenses. Treatment for OPC patients typically involves long-term drug maintenance and regular follow-up examinations, and the introduction of the CRP technique may reduce the need for subsequent treatments through early tumor control, thus offering potential economic advantages.

Several limitations should be emphasized. First, this was a retrospective single-center cohort study. Treatment allocation was based on the treatment actually received and may have been influenced by clinical decision-making, patient preference, surgical eligibility, performance status, comorbidity burden, organ function, tumor burden, metastatic distribution, and physician assessment. Therefore, selection bias and group allocation bias cannot be excluded. Although baseline characteristics were compared between groups, unmeasured confounders may still have influenced the observed differences in treatment response and PFS. Patients selected for CRP may have had more favorable operative risk profiles or better general condition than those treated with pharmacotherapy alone. Second, no formal a priori sample size calculation was performed, and the limited sample size may reduce statistical power and generalizability. Third, patients with incomplete clinical records or insufficient follow-up data were excluded, and no statistical imputation was performed, which may have introduced additional bias. Fourth, the 24-month follow-up period was relatively short, and no deaths occurred during follow-up; therefore, long-term OS could not be evaluated. Finally, complete individual patient-level time-to-event data were not preserved, limiting the ability to reliably calculate hazard ratios, 95% confidence intervals, and complete number-at-risk values for the Kaplan–Meier analysis. Accordingly, the findings should be interpreted as exploratory andhypothesis-generating. Larger prospective multicenter studies with standardized treatment allocation, adequate sample size calculation, longer follow-up, and appropriate adjustment for confounding are required to confirm the potential benefit and safety of CRP combined with pharmacotherapy in patients with OPC.

## 5. Conclusion

In conclusion, cytoreductive radical prostatectomy combined with intensified systemic therapy may be associated with improved short-term disease control and progression-free survival in selected patients with oligometastatic prostate cancer. However, given the retrospective design, limited sample size, potential selection bias, and residual confounding, these findings should be interpreted cautiously and should not be considered sufficient to support widespread clinical application. Larger prospective studies are required to confirm the potential benefit and safety of this treatment strategy.

## Author contributions

**Conceptualization:** Bai Li, Qin Qin, Jinyu Liao, Li Li, Xianlong Wang, Peng Hao, Aiping Zhang, Guoqing Zhang, Chao Huang.

**Data curation:** Bai Li, Qin Qin, Jinyu Liao, Li Li, Xianlong Wang, Peng Hao, Aiping Zhang, Guoqing Zhang, Chao Huang.

**Formal analysis:** Bai Li, Qin Qin, Jinyu Liao, Li Li, Xianlong Wang, Peng Hao, Aiping Zhang, Guoqing Zhang, Chao Huang.

**Funding acquisition:** Chao Huang.

**Writing – original draft:** Chao Huang.

**Writing – review & editing:** Chao Huang.
